# Autoantibodies to Agrin in Myasthenia Gravis Patients

**DOI:** 10.1371/journal.pone.0091816

**Published:** 2014-03-14

**Authors:** Bin Zhang, Chengyong Shen, Beverly Bealmear, Samia Ragheb, Wen-Cheng Xiong, Richard A. Lewis, Robert P. Lisak, Lin Mei

**Affiliations:** 1 Department of Neuroscience and Regenerative Medicine and Department of Neurology, Medical College of Georgia, Georgia Regents University, Augusta, Georgia, United States of America; 2 Department of Physiology, Basic Medical School, Tongji Medical College, Huazhong University of Science & Technology, Wuhan, Hubei Province, P. R. China; 3 Department of Neurology, School of Medicine, Wayne State University, Detroit, Michigan, United States of America; 4 Department of Biomedical Sciences, Oakland University William Beaumont School of Medicine, Rochester, Michigan, United States of America; 5 Department of Neurology, Cedars-Sinai Medical Center, West Hollywood, California, United States of America; 6 Department of Immunology and Microbiology, School of Medicine, Wayne State University, Detroit, Michigan, United States of America; 7 Charlie Norwood VA Medical Center, Augusta, Georgia, United States of America; University of Michigan, United States of America

## Abstract

To determine if patients with myasthenia gravis (MG) have antibodies to agrin, a proteoglycan released by motor neurons and is critical for neuromuscular junction (NMJ) formation, we collected serum samples from 93 patients with MG with known status of antibodies to acetylcholine receptor (AChR), muscle specific kinase (MuSK) and lipoprotein-related 4 (LRP4) and samples from control subjects (healthy individuals and individuals with other diseases). Sera were assayed for antibodies to agrin. We found antibodies to agrin in 7 serum samples of MG patients. None of the 25 healthy controls and none of the 55 control neurological patients had agrin antibodies. Two of the four triple negative MG patients (i.e., no detectable AChR, MuSK or LRP4 antibodies, AChR-/MuSK-/LRP4-) had antibodies against agrin. In addition, agrin antibodies were detected in 5 out of 83 AChR+/MuSK-/LRP4- patients but were not found in the 6 patients with MuSK antibodies (AChR-/MuSK+/LRP4-). Sera from MG patients with agrin antibodies were able to recognize recombinant agrin in conditioned media and in transfected HEK293 cells. These sera also inhibited the agrin-induced MuSK phosphorylation and AChR clustering in muscle cells. Together, these observations indicate that agrin is another autoantigen in patients with MG and agrin autoantibodies may be pathogenic through inhibition of agrin/LRP4/MuSK signaling at the NMJ.

## Introduction

Autoimmune MG is the most common disorder of NMJ, affecting nearly 20 per 100,000 people in various populations [1]–[5]. MG patients show characteristic fatiguing weakness of voluntary ocular, bulbar and limb muscles, dysarthria, dysphagia and in severe cases death from difficulty with breathing. The symptoms and pathology of MG are known to be due to an antibody-mediated, autoimmune attack directed against molecules at the NMJ. Autoantibodies against AChR can be detected in the circulation of ∼80–90% of MG patients [6], [7]. Evidence from classic experiments indicates the anti-AChR antibodies are pathogenic [8]–[11].

However, AChR antibodies cannot be detected in ∼10–20% of generalized MG patients. Recent studies shed light on understanding the pathology in these “seronegative” MG. Approximately 40–70% of the seronegative patients have antibodies against MuSK [4], [5], [12]–[15]. Our group and others also reported that 2–50% of AChR and MuSK double seronegative patients have anti-LRP4 antibodies [16]–[19].

However, in at least 2–5% of MG patients identifiable antibodies to a known autoantigen have not been detected. The NMJ is a cholinergic synapse that rapidly conveys signals from motoneurons to muscle cells [20]–[26]. Previous studies suggest a critical role of the agrin/LRP4/MuSK pathway in formation of the NMJ. Neuronal agrin is a large extracellular matrix protein utilized by motoneurons to induce AChR clustering and postjunctional differentiation [27]–[32]. Agrin binds to LRP4 to form a tetrameric complex, which interacts with and activates MuSK to initiate downstream signaling cascades mediating AChR clustering [33], [34]. Ablation of the genes encoding for agrin, MuSK or LRP4 prevents NMJ formation [35]–[41]. We posit that agrin may be a potential autoantigen for its function at the NMJ and spatial proximity with AChR, MuSK and LRP4.

Here we show that approximately 50% of known triple seronegative MG patients (i.e., no detectable AChR, MuSK or LRP4 antibodies, AChR-/MuSK-/LRP4-) have serum antibodies against agrin, representing approximately 2–3% of all MG patients in our study. The agrin autoantibodies recognized agrin protein expressed in transfected HEK293 cells and inhibited agrin-induced AChR clustering in cultured myotubes. Our results indicate the potential involvement of agrin antibody in the pathogenesis of AChR/MuSK/LRP4-seronegative MG, thus defining one novel immunological form of the disease. Measurement of agrin antibodies would also substantially aid diagnosis and clinical management. In addition, agrin antibodies are also found in the serum of patients with antibodies to other components of the NMJ such as AChR, although not to date in our studies in those with MuSK antibodies. Studies of those patients might contribute to understanding the pathogenic mechanisms of the disease.

## Materials and Methods

### Ethics statement

Serum samples from Wayne State University were all archival and had been previously collected as part of prior Wayne State University IRB approved research studies or as additional serum obtained at the time of diagnostic studies, with informed consent for all samples. All samples were anonymized.

### Patient sera

Serum of 93 patients with MG had previously been tested for anti-AChR and anti-MuSK antibodies or tested for these antibodies for this study. Additionally we tested serum of 6 patients with MG in whom we had no data on antibody status to AChR, MuSK but were known to be negative for LRP4 antibodies. All of these were negative for agrin but since we have no data on the antibody status of these sera, they have not been included in the statistical analysis. Patients and healthy volunteers gave their written informed consent. Serum samples were assayed for AChR binding antibody at ARUP Laboratories (Salt Lake City, UT; positive ≥0.5 nM/L), at the Mayo Clinic (Rochester, MN; positive >0.02 nM/L) or at Athena laboratories (≥0.5 nM/L). Anti-MuSK was either assayed by Dr. Angela Vincent as part of a multi-institutional study of serum from MG patients (positives as defined previously [12]) or by a commercial laboratory (Athena). LRP4 antibodies were examined in our previous report [16]. Seropositive MG was defined as AChR, MuSK and/or LRP4 antibodies positive. Normal control sera were obtained from age and gender-matched volunteers serving as controls of other studies of MG. In addition, sera from patients with the following diseases were examined: amyotrophic lateral sclerosis (ALS) (n = 9); chronic inflammatory demyelinating polyneuropathy (CIDP) (n = 4); primary CNS Sjogrens syndrome (n = 2); Guillain-Barre syndrome (GBS)/acute inflammatory demyelinating polyneuropathy/(AIDP) (n = 6); acute motor axonal neuropathy (AMAN) (n = 1); GBS with concomitant Isaac's syndrome (n = 1); CNS Lyme disease (n = 1); multiple sclerosis (MS) (n = 20); paraneoplastic neuropathies (n = 2); polymyositis in a patient with primary Sjogrens syndrome (n = 1); polychondritis with CNS vasculitis (n = 1); neuromyelitis optica spectrum disorder (NMOSD) (n = 1); inflammatory myelopathies (not transverse myelitis or NMO)(n = 3); peripheral neuropathy of unknown etiology (n = 1); and neuroscarcoisis (n = 1). Overall, we tested sera from 93 immunologically-characterized MG patients, including AChR+/MuSK-/LRP4- (n = 83), AChR-/MuSK+/LRP4- (n = 6) and AChR-/MuSK-/LRP4- (n = 4). There were also 4 who were previously shown to be LRP4- but of unknown status re: AChR and MuSK antibody (negative for agrin as noted in Results, these were not among the 93 in the final data analysis) and sera from normals (n = 25) and other disease controls (n = 55) as indicated above were assayed for antibodies to agrin.

### Recombinant protein production and purification

pFlag-agrin construct was described previously [42]. Of note, this construct contains 6XHis tag between Flag-tag and agrin coding sequence, enabling metal affinity chromatography for recombinant agrin protein. HEK293 cells were transfected with pFlag-agrin and 24 hr later, cells were switched to Dulbecco's Modified Eagle Medium supplemented with reduced concentration (0.5%) of fetal bovine serum. Conditioned media containing secreted agrin proteins were harvested 24 hr later and were purified by affinity chromatography using TALON Resins (BD Biosciences). Expression and purification of agrin proteins were verified by Western blot with anti-Flag antibody (Sigma).

### ELISA detection of antibodies to agrin

Maxi-Sorp Immuno 96-well Plates (Nunc) were coated with 50 μl of 1 μg/ml agrin in the coating buffer containing 50 mM carbonate (pH 9.6) at 40C overnight, washed six times with TBST (0.1% Tween 20 in 50 mM Tris, 150 mM NaCl, pH 7.6) and incubated with the blocking buffer containing 5% nonfat milk in TBST to block non-specific binding. Sera were diluted 1∶10 in the blocking buffer (100 μl per well) and incubated for 1 hr at 370C. After six washes with TBST, the wells were incubated with alkaline phosphatase (AP)-goat anti-human IgG+IgM+IgA as the secondary antibody (Abcam), diluted 1∶30,000 in TBST, at 37°C for 1 hr. Activity of immobilized AP was measured by optical density (OD) assay (at 405 nm) following incubation in the substrate buffer containing 0.5 mM MgCl2, 3 mg/ml p-nitrophenyl phosphate (pNPP) and 1 M diethanolamine (DEA), at room temperature for 30 min. Each sample was assayed in duplicate and repeated more than three times. Nonspecific signal was determined by OD reading of wells coated with the coating buffer alone followed by incubation of secondary antibody and substrate. Cut-off value was set as mean +3 standard deviation (SD) of control normal human serum, representing confidence of 99.7%.

### Immunoprecipitation of agrin by serum samples with agrin autoantibodies

Conditioned media containing Flag-agrin were incubated with 10 μl of sera (sera 1–21, 1–106, 2–17, 2–27 and normal human serum control) at 4°C overnight with agitation, followed by 2 hr incubation with 50 μl Protein-G beads at 4°C. Bead-immobilized proteins were subjected to SDS-PAGE and western blotting with anti-Flag antibody.

### Immunostaining of agrin-transfected HEK293 cells by serum samples

HEK293 cells were transfected with pFlag-agrin and 72 hr later, cells were washed briefly with PBS and fixed in 4% paraformaldehyde. After permeabilization with 0.5% Triton X-100 in PBS for 5 min, cells were blocked with blocking buffer containing 10% normal goat serum and 1% BSA in PBS. MG patient and normal human control serum samples were diluted 1∶10 in blocking buffer containing rabbit anti-Flag antibody (1∶500 dilution) and incubated with cells at 4°C overnight. After wash, FITC labeled goat anti-human IgG secondary antibody (Southern Biotech) and Alexa 594 labeled donkey anti-rabbit secondary antibody (Invitrogen) were added and incubated for 1 hr. After wash, cells were mounted and viewed under a Zeiss epifluoresence microscope. At least 5 views per dish and at least 2 dishes were scored in two independent experiments. All samples were examined blindly without previous information of the diagnosis.

### Effects of agrin positive sera on agrin-induced MuSK phosphorylation and AChR clustering

Agrin-induced MuSK phosphorylation and AChR clustering were assayed as previously described [33], [43], [44]. Briefly, C2C12 myotubes were treated with neural agrin (10 ng/ml) [33] together with agrin positive sera (1∶100 dilution) (sera 1–21, 1–106, 2–17, 2–27) or normal human control serum for 30 min. After brief wash, cells were lyzed in RIPA buffer and incubated with anti-MuSK antibody at 4°C overnight with agitation, followed by 2 hr incubation with 50 μl Protein-G beads at 4°C. Bead-immobilized proteins were subjected to SDS-PAGE and western blotting with anti-phospho-tyrosine antibody 4G10 (Millipore). For AChR clustering assay, myotubes were treated with neural agrin (10 ng/ml) together with agrin positive sera (1∶100 dilution) (sera 1–21, 1–106, 2–17, 2–27) or normal human control serum for 16 hr, then fixed in 4% paraformaldehyde, and incubated with 50 nM rhodamine-conjugated-bungarotoxin (R-BTX) (Invitrogen) to label AChR clusters. Myotubes were viewed under a Zeiss epifluoresence microscope and AChR clusters with diameters or a longer axis ≥4 μm were scored. At least 10 views per dish and at least 2 dishes were scored in each of three independent experiments.

### Statistical Analysis

For ELISA examination of control and MG patient sera, all samples were tested in triplicate in three independent experiments. The cut-off value was set as mean +3 SD of normal human serum samples to represent 99.7% confidence. For AChR clustering assay, data of multiple groups was analyzed by ANOVA, followed by a student-New-man-Keuls test. Differences were considered significant at p<0.05.

## Results

### Detection of agrin autoantibodies in sera of MG patients

**Figure 1:**
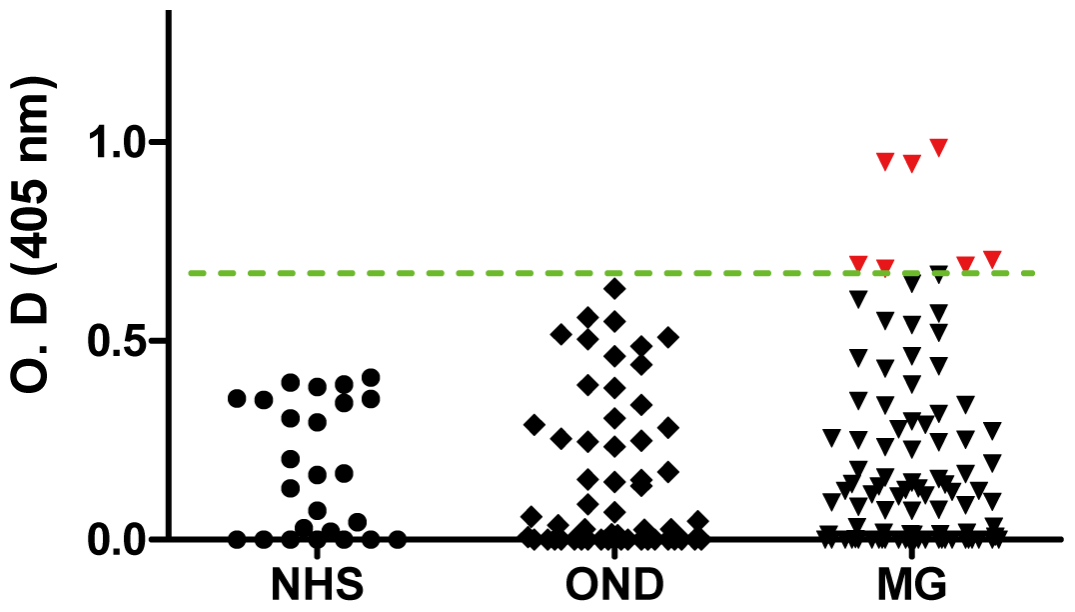
Detection of agrin autoantibodies in MG patient samples. Optical density readings of normal human serum were 0.18 ± 0.16 (mean ± SD, n  =  25). The green dotted line was set as mean + 3 SD to indicate the cut-off. The red dots indicate positive for agrin antibodies. NHS, normal human serum; OND, other neurological diseases, n  =  55; MG, myasthenia gravis, n  =  93.

**Figure 2:**
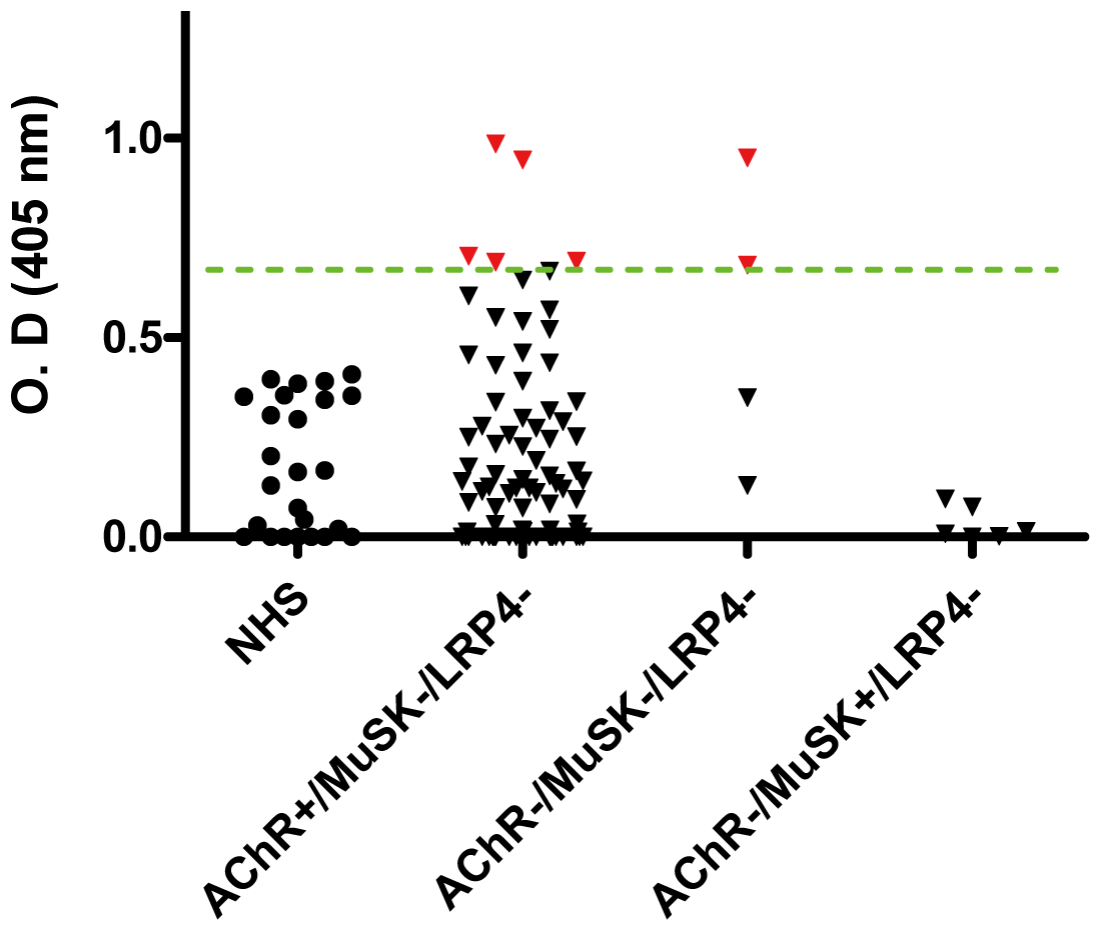
Distribution of agrin autoantibodies among MG patients. Of 93 MG samples previously analyzed for antibody to AChR, MuSK and LRP4, 83 were AChR+/MuSK-/LRP4-; 4 were triple seronegative (AChR-/MuSK-/LRP4-) and 6 were AChR-/MuSK+/LRP4-. The cut-off, indicated by the green line, was set as mean + 3 SD. The red dots indicate positive for agrin antibodies.

To determine whether sera of MG patients have agrin autoantibodies as well as to characterize those sera with regard to antibodies to other known autoantigens at the NMJ, we generated Flag/His-tagged rat agrin (His1137 to Pro1940). The purified protein resolved around 120 kDa on SDS-PAGE. Moreover, it could be detected by a commercial antibody against the Flag epitope (data not shown). The agrin protein was used in ELISA assays for autoantibodies in sera from MG patients as well as various groups of control individuals. With the mean plus 3 SD of normal sera as cut-off, none of the serum samples from normal individuals were positive for agrin antibodies. No positive sera were detected from patients with non-MG neurological disorders as defined in methods (Fig. 1). Of 93 MG patients, 7 were positive for agrin autoantibodies: 5 were AChR+, but MuSK- and LRP4- patients and 2 were from patients who were ‘triple seronegative’ (no AChR, MuSK or LRP4 antibodies) (Fig. 2). As noted earlier, there were sera from 6 patients with MG who were known to be negative for LRP4 antibodies but had not been tested for AChR and MuSK antibodies and they were all negative for agrin antibodies (data not shown in Figs. 1 or 2).

**Figure 3:**
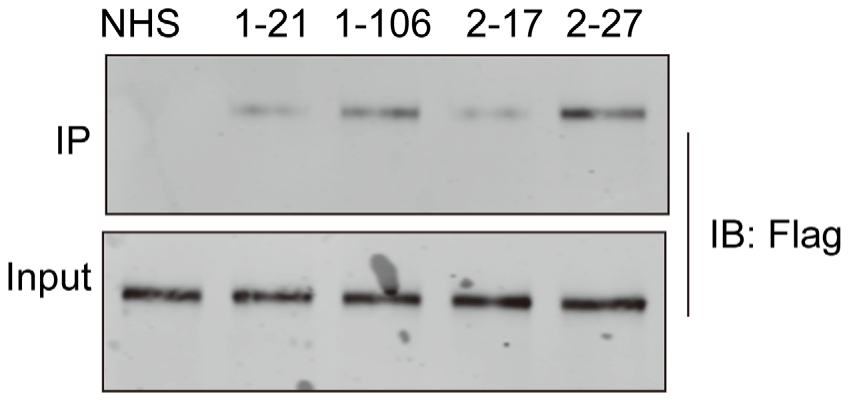
Recognition of agrin protein by serum samples with agrin autoantibodies. Conditioned media from Flag-agrin-transfected HEK293 cells were incubated with serum samples with agrin antibodies or normal human serum samples. Immunocomplex and conditioned media (to indicate equal amounts of input) were subjected to Western blotting with anti-Flag antibody.

To confirm that the target antigen of these sera was agrin, rather than any contaminant in the agrin preparation, we examined whether the agrin positive sera could recognize agrin in soluble form. Four agrin+ sera were incubated with Flag-agrin conditioned media. The immunocomplex was purified by protein G immobilized on beads, resolved by SDS-PAGE and subjected to western blot analysis with anti-Flag antibody. As expected, agrin was not detectable in the immunocomplex by normal human serum. However, Flag-tagged agrin was detected in the precipitates by 4 agrin positive sera, indicating that agrin autoantibodies were able to recognize agrin protein in solution from transfected cells (Fig. 3).

**Figure 4:**
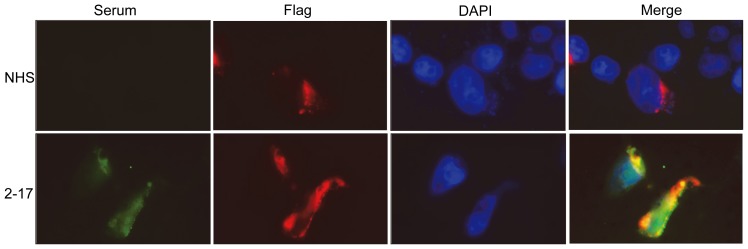
Recognition of agrin from transfected HEK293 cells by agrin+ serum. Agrin positive serum 2–17 stained positively with HEK293 cells transfected with Flag-agrin construct, co-staining with anti-Flag antibody. Normal human serum cannot recognize agrin-transfected cells.

To further confirm that the sera are able to recognize agrin in natural condition, we examined whether the agrin positive sera could detect agrin in transfected cells. One agrin positive serum (2–17) was used to stain HEK293 cells transfected with Flag-agrin construct. As expected, agrin positive serum was able to detect transfected agrin in HEK293 cells, co-staining with anti-Flag antibody. However, Flag-tagged agrin was not detected by normal human serum, indicating that agrin autoantibodies were able to recognize agrin protein in transfected cells (Fig. 4).

### Alteration of agrin-induced MuSK phosphorylation and AChR clustering by agrin autoantibodies

**Figure 5:**
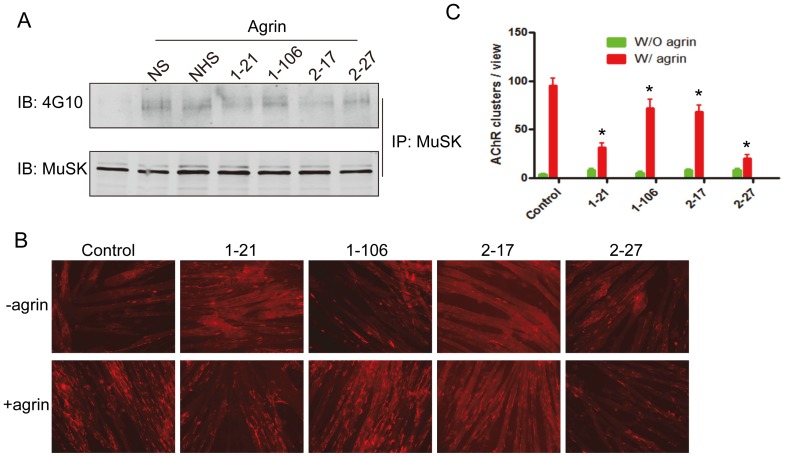
Serum samples with agrin antibodies inhibit agrin-induced MuSK phosphorylation and AChR clustering in myotubes. A, Anti-agrin autoantibodies inhibit agrin-induced MuSK phosphorylation. C2C12 myotubes were incubated without or with agrin and serum samples. Endogenous MuSK was precipitated by MuSK antibody and its phosphorylation was examined by 4G10 antibody. NS, no serum. B, Anti-agrin autoantibodies inhibit agrin-induced AChR clustering. Representative images. C, Quantitative data of basal (W/O agrin, green) and induced (W/agrin, red) AChR clusters. Data shown were mean ± SEM. *, p < 0.05, compared with control.

Agrin induces Tyrosine phosphorylation of MuSK, which is critical for downstream cascades activation and agrin-induced AChR clustering [45]. We speculated the autoantibodies may change agrin-induced MuSK phosphorylation and thus AChR clustering. To test this hypothesis, C2C12 myotubes were treated with neural agrin alone or together with control or agrin+ sera, and examined for MuSK phosphorylation and AChR clusters. As shown in Fig. 5A, neural agrin induced MuSK phosphorylation in myotubes without serum treatment or treated with normal human serum. However, the phosphorylation was decreased in anti-agrin sera treated myotubes, especially 1–21 and 2–17 samples, indicating the blocking effect of the autoantibodies on agrin signaling. In AChR clustering assay (Figs 5B and 5C), agrin-induced AChR clusters in myotubes were not altered by normal human sera, but were inhibited by all agrin positive sera. These results suggest that agrin autoantibodies may have pathogenic role through its inhibition on AChR clustering induced by agrin.

## Discussion

About 80–90% of MG patients have detectable serum antibodies against AChRs with 40–70% of the remaining patients being positive for anti-MuSK antibodies and 2–50% for anti-LRP4 antibodies [12], [16]–[18], [46]. This would leave approximately 2–5% of the MG patients triple seronegative, i.e., without detectable antibodies against any known autoantigen (AChR, MuSK or LRP4) at the NMJ. This study presents evidence that anti-agrin autoantibodies exist in sera of the triple seronegative MG patients, as well as in patients with AChR antibodies. In our cohort of 93 serologicallly characterized patients, 7 were found positive for anti-agrin antibodies, accounting for about 7–8% of all MG patients. The presence of agrin antibodies in 2 out of 4 ‘triple seronegative’ patients with MG suggests that agrin may be a novel antigen in some triple seronegative MG patients. It is worth noting that we found no agrin antibody in any of our patients who had MuSK antibodies. Since none of the 93 patients tested for agrin antibodies in this study were positive for LRP4 antibody as tested in our previous paper [16], we do not know if some LRP4+ patients will be found to have agrin antibodies in future studies.

During the preparation of this study, a group from the United Kingdom reported the detection of agrin autoantibody in seronegative MG patients [47] using a cell-based assay. They found that in triple seronegative MG patients, 15% were anti-agrin positive. Also they showed high percentage of overlapping between AChR+ and agrin+ patients (13 AChR+ in total 24 agrin+ patients). Although detailed methodology was not included, the results from the report support what we observed in current study. Due to the time consuming nature of cell-based methods, our ELISA-based assay reported here would provide a convenient yet reliable clinical diagnostic test with quantitative value.

Pathogenic mechanisms of AChR antibodies have been well studied. In rabbit, mouse, and rat models of experimental autoimmune myasthenia gravis (EAMG), anti-AChR antibodies block the activity of the AChR [48], [49] and may accelerate the internalization and degradation of AChRs [50]. In addition, the autoantibodies may fix complement, leading to complement activation causing damage and simplification of the postsynaptic membrane of the NMJ [10], [51]–[54]. The AChR deficiency decreases the amplitude of miniature end-plate potentials (mEPPs) and hence that of end-plate potentials (EPPs), which consequently reduces the safety margin of neuromuscular transmission [10], [11]. On the other hand, MuSK antibodies seem to inhibit the activity of MuSK, leading to attenuation of agrin-induced AChR clustering thus reducing AChR levels at the junctional folds [55]–[59]. In addition, NMJs and AChR scaffolds are disrupted in MuSK antibody induced EAMG. However MuSK antibodies in MG patients are predominantly of the IgG4 subclass [60], [61] which does not bind and activate complement. Thus, it seems that MuSK antibody-associated MG may have different etiological and pathological mechanisms from those of the AChR antibody associated MG. This concept is also supported by the observation that MG patients with MuSK antibodies patients do not appear to have thymic hyperplasia or thymoma [62]–[66]. The pathogenic role of LRP4 autoantibodies has been presented in the EAMG recently by our lab [67].

Whether and how agrin autoantibodies are pathogenic requires further study. We have demonstrated that some agrin+ sera were able to inhibit agrin-induced AChR clustering which provides one possible pathologic role of these antibodies in vivo. It is of note that agrin protein has multiple isoforms and can be secreted by muscle and motor neuron (muscle and neural agrin, respectively) [68]. Neural agrin has up to 1000-fold greater AChR clustering activity compared to other isoforms and was used throughout this study. However the primary sequences between neural and muscle agrin mainly differ at the Z insert, only 8 amino acids [69]. Considering the large size of agrin, it is likely that the agrin autoantibodies also recognize muscle agrin. Whether the antibodies against muscle agrin are pathogenic would also be interesting to explore.
